# Medicare Advantage Rapid Disenrollment Trends

**DOI:** 10.1093/geroni/igaf122.3502

**Published:** 2025-12-31

**Authors:** Em Balkan, Jay Shroff, David Meyers

**Affiliations:** Brown University, Providence, Rhode Island, United States; Brown University School of Public Health, Providence, Rhode Island, United States; Brown University, Providence, Rhode Island, United States

## Abstract

In 2024, 54% of Medicare beneficiaries chose to receive their benefits from private plans, known as Medicare Advantage (MA). Medicare beneficiaries can switch their coverage during Medicare’s annual Fall Open Enrollment, with new coverage effective January 1 of the following year. All MA enrollees can choose to change their coverage again during the Medicare Advantage Open Enrollment Period from January 1 through March 31. Rapid disenrollment is when a beneficiary who enrolled in a new MA plan during the fall decides to disenroll immediately during the subsequent spring. Rapid disenrollment has been severely understudied. In our analysis of national Medicare data from 2017 to 2022, we found the rate of rapid disenrollment more than tripled, from 4 to 12.2%. The rate of rapid disenrollment was highest for Medicare-Medicaid dual enrollees, with 20% of such beneficiaries rapidly disenrolling by 2022. Additionally, higher proportions of Black, Hispanic, and Native enrollees rapidly disenrolled from their MA plans compared to white enrollees. Plans serving many Medicare-Medicaid dual enrollees tended to have higher rates of rapid disenrollment. It is unclear why rapid disenrollment has increased so dramatically, indicating that beneficiaries quickly decide that their new MA plan is not the best option for them. It is also unclear why rapid disenrollment rates are higher for communities of color and those with lower incomes. For these reasons, and as MA becomes more popular, it is critical to better understand rapid disenrollment in order to improve health care access and quality for Medicare beneficiaries.

